# Breast Trauma: A United States-Based Epidemiological Study From 2016 to 2019

**DOI:** 10.7759/cureus.50334

**Published:** 2023-12-11

**Authors:** Matthew Hager, Aparajita Spencer, Adam Wegener, Hanah Lee, Michelle Fillion, James Yon

**Affiliations:** 1 General Surgery, Novant Health New Hanover Regional Medical Center, Wilmington, USA

**Keywords:** motor vehicle accident, breast, penetrating breast injury, blunt chest trauma, breast trauma, breast injury

## Abstract

Background

Breast trauma is an often under-recognized injury, especially in female polytrauma patients. The purpose of this study was to assess the prevalence of breast injuries and their association with injury severity score (ISS) in trauma patients nationally.

Method

A retrospective review was performed using data from the National Trauma Data Bank® (NTDB®) from 2016 to 2019, using all applicable International Classification of Diseases (ICD) codes for three outlined subgroups (abrasions, contusions, and open wounds/punctures/lacerations) with minors excluded. All continuous variables were tested as non-normally distributed, and all test results for continuous variables used the Kruskal-Wallis test. All categorical variables were tested using a chi-squared test.

Results

Patients with traumatic breast abrasions and contusions had a higher rate of intensive care unit (ICU) admissions (23.8%; n=395 and 25.3%; n=48, respectively) compared to patients with open wounds/punctures/lacerations (13.6%; n=205). Patients with abrasions and contusions to the breast had a significantly higher ISS compared to those with visible open wounds/punctures/lacerations (9 and 9, vs 5, p <0.001). Mortality rates were highest among patients with breast abrasions and contusions, 15% (n=213) and 14% (n=23), respectively, compared to patients with open wounds/punctures/lacerations at 11% (n=132), p<0.017.

Conclusion

Traumatic breast abrasions and contusions were associated with higher rates of ICU admission, elevated ISS, and overall mortality compared to open breast wounds, punctures, or lacerations. This indicates the importance of traumatic breast injuries as a prognostic indicator in the standard workup of a trauma patient.

## Introduction

The breast is a complex organized system of connective tissue, ducts, glands, fat, nerves, and arteries. This organ is highly vascularized, obtaining its blood supply from a variety of different blood vessels, including the internal thoracic artery, lateral thoracic artery, thoracoacromial artery, and intercostal arteries, among others. These arteries are at risk of damage, evulsion, shear, and even transection in the trauma patient. Penetrating trauma, specifically to the anterior chest, imposes a threat to the integrity of these vessels. Additionally, motor vehicle accidents (MVAs) present a unique risk, given that the shoulder restraint seatbelts generally lay over the breast creating an area of increased tension [1,2].

A large trauma series reported a reduction in deaths related to MVAs with the transition to three-point lap-diagonal seatbelts, but there has since been a shift in the injuries seen [2]. In the breast, the increased pressure applied by the diagonal strap can cause crush injury, such as ulceration and necrosis of tissue, as well as shearing or avulsion injuries [3]. During the initial survey, these injuries may be difficult to identify as bleeding can occur deep in the breast tissue or have a delayed presentation [2,3]. While transection of perforating arteries entering the breast can cause immediate hemorrhage, initial vasoconstriction of arteries or vasospasm of the underlying pectoral muscle may delay bleeding [3,4].

Breast trauma is an often under-recognized injury, particularly in female polytrauma patients, and can be a clinically relevant source of bleeding leading to significant morbidity such as hemorrhagic shock [5,6]. Reports have shown that 17% of patients with breast hematomas require interventions ranging from multiple blood transfusions, angioembolization, or operative intervention [7,8]. One single-site trauma center study found an association between female blunt breast trauma and elevated injury severity (ISS) scores [8]. This study aimed to retrospectively review data from the National Trauma Data Bank® (NTDB®) to assess the prevalence of breast injuries and their association with ISS in trauma patients. We hypothesize that patients with traumatic breast abrasions and contusions would have a higher associated ISS score compared to patients with open wounds, punctures, or lacerations. 

## Materials and methods

A retrospective review of traumatic breast injuries from January 1, 2016, to December 31, 2019, was conducted using NTDB data. All applicable International Classification of Diseases (ICD)-10 codes were used to isolate breast injuries for three outlined subgroups: (i) abrasions, (ii) contusions, and (iii) open wounds/punctures/lacerations (See Appendices). Trauma patients with breast injuries that occurred prior to January 1, 2016, and after December 31, 2019, were not included in this study. Additionally, patients with minor injuries (e.g. breast pain or numbness) that did not qualify for the categories listed above were excluded from the study. No other exclusions were applied. This study was exempt from our Institutional Review Board based on the study design and utilization of de-identified data within the NTDB.

Data source 

The NTDB is a free database comprising the largest aggregation of United States (US) trauma registry data. It contains de-identified data from adult and pediatric trauma centers (Levels I, II, III, IV, V, or undesignated) across the US, including patient demographics, injury information, and outcomes. Sponsored and supported by the American College of Surgeons, the NTDB is compiled from records submitted by US trauma centers yearly, dating back to 2004. In addition to research datasets, the NTDB provides participating hospitals with benchmark and data quality reports, among others [9]. 

Data analysis and study variables 

Within these three subgroups (abrasions, contusions, and open wounds/punctures/lacerations), patient demographics were organized into clinically meaningful categories of age, sex, race, ED disposition, mortality, and injury severity score (ISS). The frequencies of categorical variables were calculated across the three subgroups. Chi-squared test was performed on all categorical variables. Significance was defined as two-tailed p < 0.05. All continuous variables were tested as non-normally distributed. All test results for continuous variables were calculated using the Kruskal-Wallis test. All analysis was performed with R 4.1.2 statistical programming software (R Foundation for Statistical Computing, Vienna, Austria).

## Results

Demographics 

There were 3,387 traumatic breast injuries in the study period in the US according to the NTDB within the ICD codes specified. This study comprised 72% female (n=2,474) and 27% male (n=913) subjects. A total of 719 out of the 3,387 identified race, of which 57% (n=413) of subjects identified as White. Black or African American patients made up roughly 30% (n=213) of patients who identified race. The median age of patients who experienced a traumatic abrasion to the breast was 38 years. Patients who experienced a contusion were significantly older with a median age of 47 years. Lastly, the median age of patients with an open wound/puncture/laceration was 34 years. Individuals who identified as White were noted to have the majority of traumatic breast abrasions (71%, n=258, p<0.001). Conversely, Black or African American patients comprised the majority of the open wound/puncture/laceration group (46%, n=151, p<0.001) (Table 1). 

**Table 1:** Patient Demographics and Injury Patterns Among Traumatic Breast Injuries The data has been represented as n, the number of trauma patients, as well as the percentage (%) of trauma patients within the three subgroups: Abrasions, Contusions, and Open Wounds/Punctures/Lacerations, unless otherwise mentioned. Significance is defined as p<0.05.

	Abrasions (n=1675)	Contusions (n=193)	Open Wounds/Punctures/Lacerations (n=1520)	p-value
Age (years), median (IQR)	38 (26, 55)	47 (31, 62)	34 (26, 47)	<0.001
Sex, n (%)				<0.001
Female	1210 (72.3%)	165 (85.5%)	1099 (72.3%)	
Male	464 (27.7%)	28 (14.5%)	421 (27.7%)	
Race, n (%)				<0.001
American Indian	1 (0.3%)	0 (0.0%)	3 (0.9%)	
Asian	5 (1.4%)	0 (0.0%)	5 (1.5%)	
Black or African American	56 (15.5%)	6 (19.4%)	151 (46.3%)	
Native Hawaiian or Other Pacific Islander	2 (0.6%)	0 (0.0%)	0 (0.0%)	
Other Race	40 (11.0%)	2 (6.5%)	35 (10.7%)	
White	258 (71.3%)	23 (74.2%)	132 (40.5%)	
ED Disposition, n (%)				<0.001
Deceased	156 (9.4%)	15 (7.9%)	100 (6.6%)	
Home	153 (9.2%)	11 (5.8%)	229 (15.2%)	
Hospital	719 (43.2%)	83 (43.7%)	499 (33.2%)	
ICU	395 (23.8%)	48 (25.3%)	205 (13.6%)	
Operating Room	188 (11.3%)	27 (14.2%)	420 (27.9%)	
Other	9 (0.5%)	1 (0.5%)	24 (1.6%)	
Transferred	43 (2.6)	5 (2.6)	28 (1.9)	
ISS, median (IQR)	9 (5, 14)	9 (5, 17)	5 (1, 14)	<0.001
Mortality, n (%)				0.017
No	1227 (85.2%)	147 (86.5%)	1064 (89.0%)	
Yes	213 (14.8%)	23 (13.5%)	132 (11.0%)	

ED disposition 

**Figure 1:**
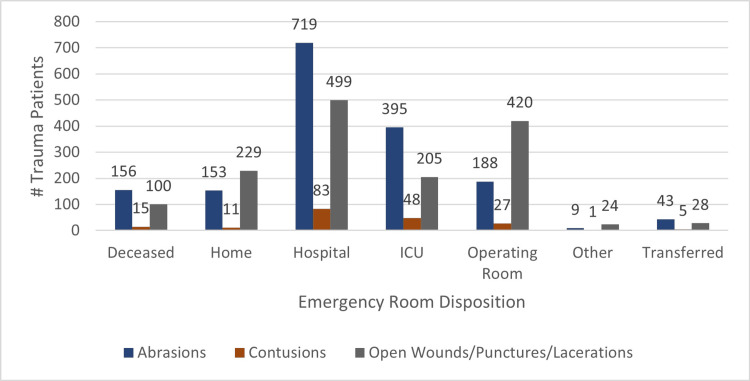
ED Disposition of Patients with Traumatic Breast Injuries The data has been represented as n, the number of trauma patients within the three subgroups: Abrasions, Contusions, and Open Wounds/Punctures/Lacerations.

The majority of patients within the three subgroups (abrasions, contusions, and open wounds/punctures/lacerations) were admitted to the hospital; 43% (n=719), 44% (n=83), and 33% (n=499), respectively. Of trauma patients with open wounds/punctures/lacerations of the breast, 15% (n=299) were discharged home. Patients with abrasions and contusions had a higher rate of ICU admissions (23.8%; n=395 and 25.3%; n=48, respectively) compared to patients with open wounds/punctures/lacerations (13.6%; n=205). Of patients with open wounds/punctures/lacerations, 28% (n=420) were taken to the operating room from the ED, compared to 11% (n=188) of abrasions, and 14% (n=27) of contusions (p<0.0010) (Figure 1).

Mortality and ISS

**Figure 2:**
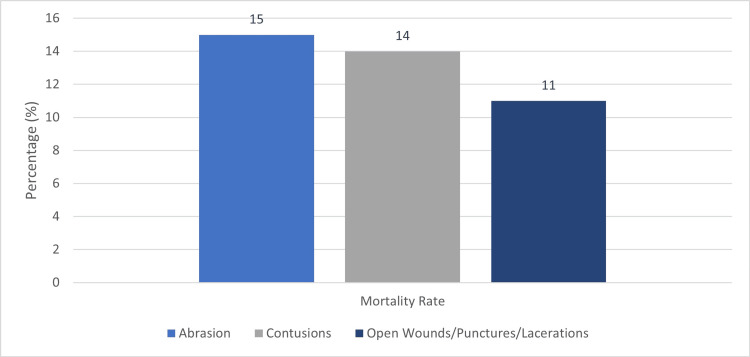
Mortality Rates Among Patients with Traumatic Breast Injuries The data has been represented as the percentage (%) of mortality among trauma patients within the three subgroups: Abrasions, Contusions, and Open Wounds/Punctures/Lacerations.

Breast trauma patients with abrasions had the highest rate of mortality at 15% (n=213), compared to 14% (n=23) of those with contusions, and 11% (n=132) of those with open wounds/punctures/lacerations (p<0.017) (Figure 2). In addition, patients with abrasions and contusions to the breast were more likely to have a significantly higher ISS compared to those with visible open wounds/punctures/lacerations (9 and 9, respectively, vs 5, p < 0.001).

## Discussion

With the rapid expansion of registered trauma centers in the US, this work represents the most up-to-date epidemiological study regarding injury patterns and disposition of traumatic breast injuries. Of the 3,387 trauma patients, the majority were females (72%; n=2474) and those who identified as White (57%; n=413). The median age of our sample ranged from 34 years in the open wound/puncture/laceration group to 47 years in the contusion group. Of the charts that identified race (719 out of the 3,387), Black or African American patients comprised the majority of the open wound/puncture/laceration group (Table 1). These sample characteristics are similar to previous epidemiologic and self-reporting studies [6-8]. Although not specified in this study, the majority of traumatic breast injuries occur from MVAs [5-8,10-13].

The injury subgroups within this study were categorized as abrasion, contusion, and open wounds/punctures/lacerations, which differs from many previous studies that focused solely on breast hematomas [5,7]. These subgroups were categorized in this fashion so that the practitioner would be able to distinguish the injury upon primary assessment, before any radiographic utilization. The injury classifications in this study are more specific but are similar to the categories of blunt vs penetrating injuries seen within similar studies [8,10]. 

Trauma patients with abrasions and contusions had a higher rate of hospitalization and ICU admission compared to patients with an open wound/puncture/laceration (Figure 1, Table 1). ISS for the abrasion and contusion groups were also significantly higher than the open wound/puncture/laceration group (9 and 9, respectively, vs 5) (p < 0.001). This data reveals that trauma patients with a traumatic breast abrasion or contusion have, on average, a higher likelihood of having additional comorbidities. This is consistent with the findings of Sargent et al. in which ISS were highest after blunt breast trauma compared to penetrating [10]. Sanders et al. reported in a retrospective analysis of female blunt breast trauma patients that the most common associated comorbidities included long bone fractures (45%) and rib fractures (44%) [8]. Both of these injuries are associated with elevated ISS scoring and increased risk of ICU admission [14]. It is more likely that the traumatic breast injuries seen in this study are correlated with, and not the cause of, an elevated ISS and increased risk of ICU admission.

Interestingly, the open wound/puncture/laceration group had a higher rate of operative intervention at 28% (n=420), compared to the abrasion and contusion groups at 11% (n=188) and 14% (n=27), respectively. Operative intervention in this study included breast and non-breast-related procedures and therefore points to the overall severity of illness associated with the trauma patient rather than the severity of the breast injury. In consideration of operative interventions specific to breast injuries, Sanders et al. proposed a tiered algorithm for simple and complex breast injuries. Simple breast injuries, defined as an abrasion, small laceration, or pain over the affected breast, were managed conservatively. Patients with a complex breast injury, defined as a crush injury to the breast that causes an intramammary hematoma or tissue loss, that were hemodynamically stable underwent a computed tomography (CT) scan with intravenous contrast of their chest. Those patients with arterial extravasation underwent angiography and embolization, while patients who did not have extravasation were monitored and treated symptomatically [8]. Despite this, angiographic intervention in patients with arterial extravasation of the breast remains controversial [15,16]. Patients with complex breast injuries that were hemodynamically unstable went to the operating room for laceration repair, mastotomy, or in some cases, mastectomy [10].

Mortality rates were highest for patients with traumatic abrasions and contusions at 15% (n=213) and 14% (n=23), respectively, compared to 11% (n=132) of patients with open wounds, punctures, and/or lacerations. Sargent et al. reported a similar trend in their retrospective review, with higher mortality rates and injury severity scores among blunt breast trauma patients compared to penetrating injuries [10]. Although, this data did differ from one single-site retrospective review of 871 patients with traumatic breast hematomas that reported an in-hospital mortality rate of 0%, previous studies using the NTDB revealed higher mortality rates [7,10]. Our data supports the findings of Sanders et al., in that patients with abrasions and contusions were associated with higher ISS and mortality rates compared to open wounds, punctures, or lacerations [8]. This data is significant for practicing clinicians to correlate the associated increased injury severity and mortality that accompany traumatic breast abrasions and contusions.

Limitations

The NTDB is a database that collects patient demographics and injury patterns from trauma centers throughout the US. The accuracy of patient demographics and types of injuries is limited to the correct documentation within patient charts from the selected facilities [9]. Although the number of recognized trauma centers is continuing to grow nationwide, there likely remain many patients who present to non-trauma medical facilities, such as primary care centers, urgent cares, or non-trauma recognized emergency departments/hospitals. These patients may present with less severity of traumatic breast injuries and therefore are not included in the NTDB. As such, the data in this study could overestimate the severity of illness associated with traumatic breast injuries. In addition, the NTDB does not account for pre-hospital deaths or patients who did not seek medical advice. Therefore, the correlated mortality rate may not accurately represent the true number of deaths secondary to, or with, traumatic breast injuries. Additionally, patients with prior breast implants, reconstruction, or irradiation to the breast were not detailed in this study. As such, traumatic breast injuries in this population may have led to earlier intervention, altering our results. Patient confounders such as comorbidities were also not specified in this study and therefore were not excluded from statistical analysis. However, the results of this study do support previous similar studies pointing to the increased accuracy of the data despite the inclusion of confounders [8,10]. Our results are limited to the NTDB, which was not specifically designed to study the breadth of breast-specific injuries and thus may not fully represent the true injury burden.

## Conclusions

This work represents the most up-to-date epidemiological study regarding injury patterns and disposition of breast trauma patients presenting to US trauma centers. Traumatic breast abrasions and contusions were associated with higher rates of ICU admission, elevated ISS, and overall mortality compared to open breast wounds, punctures, and/or lacerations. This indicates the importance of recognizing traumatic breast injuries as a prognostic indicator in the standard workup of a trauma patient.

## Appendices

**Table 2:** International Classification of Diseases-10 Codes for Traumatic Breast Injuries

Contusion	Abrasion	Foreign Body	Open Wound	Traumatic Injury
S20.0	S20.11	S20.15	S21.0	S28.2
S20.00	S20.111	S20.151	S21.00	S28.21
S20.00XA	S20.111A	S20.151A	S21.001	S28.211
S20.00XD	S20.111D	S20.151D	S21.001A	S28.211A
S20.00XS	S20.111S	S20.151S	S21.001D	S28.211D
S20.01	S20.112	S20.152	S21.001S	S28.211S
S20.01XA	S20.112A	S20.152A	S21.002	S28.212
S20.01XD	S20.112D	S20.152D	S21.002A	S28.212A
S20.01XS	S20.112S	S20.152S	S21.002D	S28.212D
S20.02	S20.119	S20.159	S21.002S	S28.212S
S20.02XA	S20.119A	S20.159A	S21.009	S28.219
S20.02XD	S20.119D	S20.159D	S21.009A	S28.219A
S20.02XS	S20.119S	S20.159S	S21.009D	S28.219D
S20.1			S21.009S	S28.219S
S20.10			S21.01	S28.22
S20.101			S21.011	S28.221
S20.101A			S21.011A	S28.221A
S20.101D			S21.011D	S28.221D
S20.101S			S21.011S	S28.221S
S20.102			S21.012	S28.222
S20.102A			S21.012A	S28.222A
S20.102D			S21.012D	S28.222D
S20.102S			S21.012S	S28.222S
S20.109			S21.019	S28.229
S20.109A			S21.019A	S28.229A
S20.109D			S21.019D	S28.229D
S20.109S			S21.019S	S28.229S
			S21.02	
			S21.021	
			S21.021A	
			S21.021D	
			S21.021S	
			S21.022	
			S21.022A	
			S21.022D	
			S21.022S	
			S21.029	
			S21.029A	
			S21.029D	
			S21.029S	
			S21.03	
			S21.031	
			S21.031A	
			S21.031D	
			S21.031S	
			S21.032	
			S21.032A	
			S21.032D	
			S21.032S	
			S21.039	
			S21.039A	
			S21.039D	
			S21.039S	
			S21.04	
			S21.041	
			S21.041A	
			S21.041D	
			S21.041S	
			S21.042	
			S21.042A	
			S21.042D	
			S21.042S	
			S21.049	
			S21.049A	
			S21.049D	
			S21.049S	
